# Gradient-Based Multi-Objective Feature Selection for Gait Mode Recognition of Transfemoral Amputees

**DOI:** 10.3390/s19020253

**Published:** 2019-01-10

**Authors:** Gholamreza Khademi, Hanieh Mohammadi, Dan Simon

**Affiliations:** Department of Electrical Engineering and Computer Science, Cleveland State University, Cleveland, OH 44115, USA; h.mohammadi17@csuohio.edu (H.M.); d.j.simon@csuohio.edu (D.S.)

**Keywords:** user intent recognition, transfemoral prosthesis, multi-objective optimization, biogeography-based optimization

## Abstract

One control challenge in prosthetic legs is seamless transition from one gait mode to another. User intent recognition (UIR) is a high-level controller that tells a low-level controller to switch to the identified activity mode, depending on the user’s intent and environment. We propose a new framework to design an optimal UIR system with simultaneous maximum performance and minimum complexity for gait mode recognition. We use multi-objective optimization (MOO) to find an optimal feature subset that creates a trade-off between these two conflicting objectives. The main contribution of this paper is two-fold: (1) a new gradient-based multi-objective feature selection (GMOFS) method for optimal UIR design; and (2) the application of advanced evolutionary MOO methods for UIR. GMOFS is an embedded method that simultaneously performs feature selection and classification by incorporating an elastic net in multilayer perceptron neural network training. Experimental data are collected from six subjects, including three able-bodied subjects and three transfemoral amputees. We implement GMOFS and four variants of multi-objective biogeography-based optimization (MOBBO) for optimal feature subset selection, and we compare their performances using normalized hypervolume and relative coverage. GMOFS demonstrates competitive performance compared to the four MOBBO methods. We achieve a mean classification accuracy of 97.14%±1.51% and 98.45%±1.22% with the optimal selected subset for able-bodied and amputee subjects, respectively, while using only 23% of the available features. Results thus indicate the potential of advanced optimization methods to simultaneously achieve accurate, reliable, and compact UIR for locomotion mode detection of lower-limb amputees with prostheses.

## 1. Introduction

Prosthetic legs have significantly enhanced the lifestyle of individuals with a transfemoral amputation. Prostheses help lower-limb amputees regain their walking mobility for activities such as level walking, stair ascent and descent, incline walking, sitting and standing, etc. One active research area is the development of a functional control system for each walking task [1,2,3]. The main design objective is to enable amputees to achieve walking that is similar to that of able-bodied persons, while minimizing metabolic energy expenditure. Challenges include recognizing gait modes automatically, selecting the appropriate control system corresponding to the identified gait mode, and achieving a smooth transition in real time. Activity mode recognition must be achieved in parallel with control system development to address these problems. Activity mode recognition is referred to as high-level control, while control system design for each walking activity is referred to as low-level control [4]. The focus of this paper is the development of a high-level control system.

In the design of an intent recognition system, several questions arise, including which input signals and machine learning algorithms will provide a UIR system with fast and reliable prediction performance. Previous research has addressed these questions in different ways. For instance, surface electromyography (sEMG) signals were used to train UIR [5,6]. Although sEMG resulted in high classification accuracy, Ref. [7] reported uncertain performance due to sEMG signal variability in real-world conditions. Variation could be because of electrode shift [8], skin temperature change [9], or muscle volume change [10]. Therefore, external sensors on the prosthesis have received significant attention. For instance, classifiers have been trained with data collected from mechanical sensors [11], optical distance sensors [12], and inertial measurement units [7]. In addition, Refs. [13,14] showed that the fusion of sensory measurements could enhance learning, although the amputee subject could be inconvenienced by wearing additional sensors. Various supervised machine learning algorithms have been implemented to build UIR systems, including linear discriminant analysis (LDA) [15], quadratic discriminant analysis (QDA) [16], Gaussian mixture models (GMMs) [11], support vector machines (SVMs) [14], and artificial neural networks (ANNs) [5]. To avoid the need for user-specific classifier training, Ref. [17] proposed a user-independent UIR system in which classifier performance is robust to user-specific characteristics.

Current UIR systems have been designed with one goal in mind: highest possible prediction accuracy. In clinical applications, it is extremely important that UIR can accurately predict activity modes with substantially different characteristics because misclassification can cause a loss of balance [7,18]. However, there remains a gap in the design of UIR with low complexity. UIR has low complexity if it can be implemented with only significant features extracted from minimal sensing hardware. UIR with low complexity is important because such systems: (1) eliminate unneeded body-worn sensors that may be irritating and cumbersome; (2) avoid numerical instability and overfitting during training; (3) are robust to noisy measurement signals and sensor failures; and (4) decrease computational effort, which is important for real-time operation. These reasons have motivated previous research to apply feature selection to design UIR with low complexity [7,19]. They used sequential forward selection to obtain a subset with only the most informative features. In contrast, in this paper, we develop a new framework for UIR that *simultaneously* achieves maximum accuracy and minimum complexity. Complexity and accuracy are two conflicting objectives. To the best of our knowledge, this paper is the first attempt to find a compromise solution for this problem using multi-objective optimization.

The main contributions of this paper are two-fold: (1) a new MOO method called GMOFS for optimal feature subset selection; and (2) the application of four evolutionary MOBBO methods for the UIR problem, including vector evaluated BBO (VEBBO), non-dominated sorting BBO (NSBBO), niched Pareto BBO (NPBBO), and strength Pareto BBO (SPBBO). We have chosen to use BBO in this paper as the evolutionary algorithm (EA) because of its demonstrated effectiveness and recent popularity for optimizing real-world problems [20,21]. MOBBO methods have the potential to find the global optimum [22,23]; however, they are computationally expensive due to the many required fitness function evaluations. To avoid this drawback, we propose GMOFS for feature selection.

Several different types of feature selection methods have been proposed. *Filter* methods are feature selection methods that assess the quality of a subset of features independently or with respect to the output class [24]. *Wrapper* methods are feature selection methods that assess the quality of a subset of features by measuring the prediction accuracy of a classifier that is trained with that subset [25]. *Embedded* methods are feature selection methods that overcome the disadvantages of filter and wrapper methods. Unlike filter methods, embedded methods account for the bias of the classifier, and, unlike wrapper methods, they are computationally efficient [26,27]. Various embedded feature selection algorithms have been proposed, mostly for linear problems with a single objective [27,28]. Embedded methods also incorporate regularization algorithms, such as ridge regression [29], least absolute shrinkage and selection [30], and elastic nets [31].

GMOFS is our newly proposed embedded method that simultaneously performs feature selection and classification, and that accounts for multiple objectives in nonlinear systems such as UIR. GMOFS incorporates an elastic net in multilayer perceptron (MLP) neural network training. The elastic net uses a Lagrange multiplier with a complexity parameter to reduce the feature set to an optimal subset, and the MLP classifier is trained with the optimal subset. We investigate the influence of the complexity parameter on the solution of the constrained MLP optimization problem. We then use the optimization solutions to obtain a GMOFS Pareto front, which is a set of non-dominated solutions that are equally important apart from the designer’s subjective preference of objectives.

Section 2 presents a general framework for UIR. In Section 2.1, an informative set of signals reflecting various walking tasks are collected experimentally from three able-bodied and three amputee subjects. In addition, the data are filtered and processed to eliminate noise and missing data points. In Section 2.2, we use both disjoint windowing and overlapped windowing to extract data frames. The length of the data frame and the increment of the moving window are chosen to compromise the informativeness of the data and the computational effort, while taking real-time computational constraints into account. In Section 2.3, various time-domain (TD) and frequency-domain (FD) features are extracted from each data frame for each measurement signal. A training data set is obtained in which all features are normalized to have a zero mean and unity variance. In Section 2.4, we use a pre-selection approach to exclude insignificant features, and then apply MOO for final feature selection. We implement GMOFS and four variants of MOBBO to minimize the size of the selected feature subset and maximize the prediction accuracy. In Section 2.5, the performance of several classifiers, including LDA, QDA, SVMs with both linear and radial basis function (RBF) kernels, MLPs, and decision trees (DTs), are compared, and the best one is selected for UIR. In Section 2.6, majority voting filter (MVF) is implemented to avoid sudden jumps between identified classes and enhance UIR performance. Section 4 discusses the experimental setup and classification results for the optimally designed UIR system. Finally, Section 5 discusses conclusions and future work.

## 2. Materials and Methods

**Figure 1:**
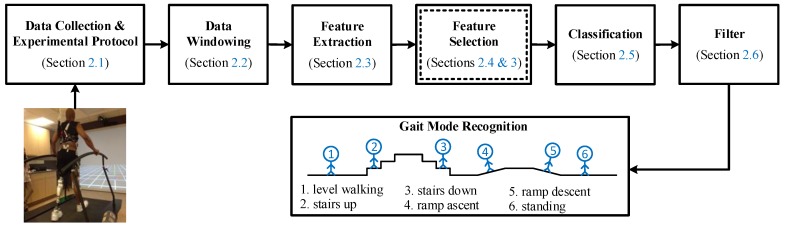
Architecture of user intent recognition system. The double-lined box indicates that an evolutionary algorithm is used for optimization.

In this section, we present the methodology used to design the user intent recognition (UIR) system. The architecture of the UIR system is illustrated in Figure 1. Our new contribution is a novel feature selection method based on multi-objective optimization (MOO), as illustrated in Figure 1 in the double-lined box (Section 2.4). In this box, the application of four multi-objective biogeography-based optimization (MOBBO) methods for gait mode classification is new, and a novel MOO-based feature selection method called gradient-based multi-objective feature selection (GMOFS) is new. The remaining parts of the UIR system are implemented based on the existing literature. The role of each subsystem is explained in more detail in the following subsections.

### 2.1. Data Collection and Experimental Protocol

Data collection can significantly affect the accuracy of UIR. Input signals must be informative enough to accurately discriminate between various human gait modes. In this paper, we collect vertical hip position and thigh angle to indicate the state of the residual limb, and thigh moment to indicate the user–prosthesis interaction. These signals are like an implicit communication link between the user and the prosthesis and can be used to infer user intent.

To design and evaluate the performance of the UIR system, we collect these signals from three able-bodied subjects (AB01, AB02, and AB03) and three transfemoral amputee subjects (AM01, AM02, and AM03). All the experiments were approved by the Department of Veterans Affairs Institutional Review Board. The above-knee amputees wore an Ottobock prosthesis on the right leg. Data were collected for able-bodied subjects during four different activity modes: (1) standing (ST), (2) normal walking (NW) at user-preferred speed (PS), (3) slow walking (SW), and (4) fast walking (FW). We asked subjects to walk slower and faster than their normal walking speed for SW and FW modes, respectively, allowing them to choose comfortable velocities for these two modes. Due to physical limitations, we collect data during only three activity modes for the amputee subjects: ST, NW, and SW. Table 1 shows the physical characteristics of the subjects.

**Figure 2:**
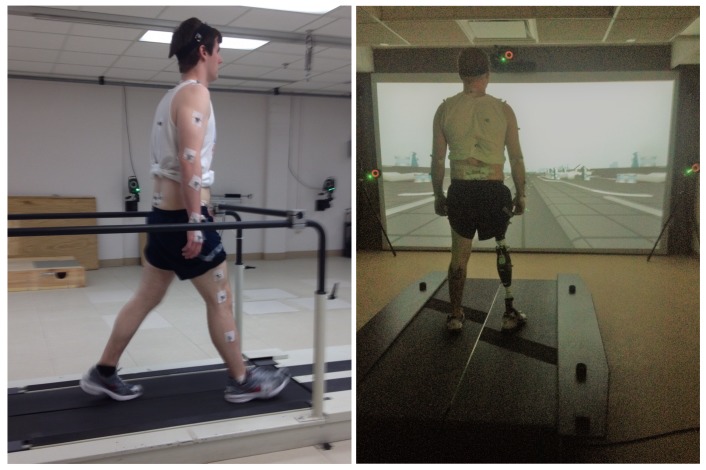
Experimental setup for data collection. The **left** figure shows an able-bodied subject and the **right** figure shows an amputee subject with an Ottobock prosthesis on the right leg.

The data were collected at the Motion Study Laboratory of the Cleveland Department of Veterans Affairs Medical Center with 47 reflective markers on each subject’s body. Subjects were asked to walk on a treadmill with built-in force sensors. A 16-camera Vicon system (Denver, CO, USA) recorded kinematic data at 100 Hz. Ground reaction force along three axes were collected from the force sensors at 1000 Hz. Data were filtered with a second-order low-pass filter with a cutoff frequency of 6 Hz. A 3D biomechanical rigid body model was constructed from the marker data, and segmental and joint kinematics (joint displacements) and kinetics (joint moments) were computed as inputs for the UIR system. Detailed methods and sample results can be found in [32]. The experimental setup is illustrated in Figure 2. Note that the lower-limb amputee demographic at the Veterans Affairs Medical Center, where data collection was performed, is dominated by males. Over 98% of veterans who underwent amputation in 2011 were male [33]. Our future work will need to include more subjects and wider demographics (for example, ages and genders).

Note that, in the real-world, non-laboratory settings, we would measure the required input signals directly rather than with cameras. For example, we could use piezo-electric sensors or multi-axis load cells for force sensing, and optical encoders or inertial measurement units for accurate position and angle sensing during stance and swing phases [7,11].

**Figure 3:**
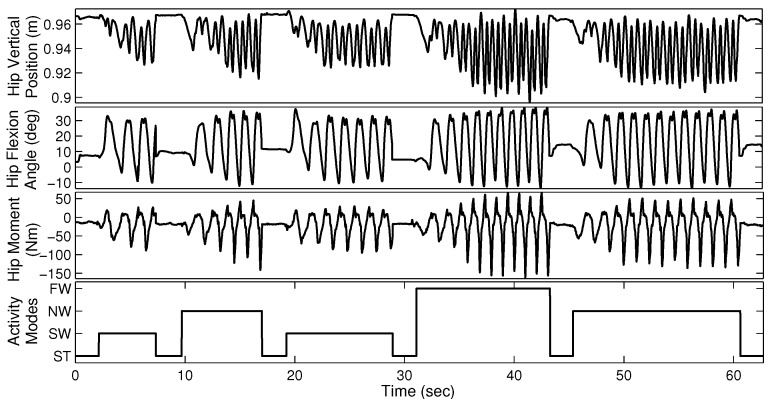
Sample walking trial with four different gait modes for able-bodied subject AB01. Although data is available for both legs, we require only one side for gait mode recognition. The data from the two legs look similar because of gait symmetry.

The able-bodied subjects were asked to perform four sequences of walking trials, each lasting approximately 60 s. Each sequence consists of four different gait modes (ST, SW, NW, and FW) and each mode was maintained for several seconds. Figure 3 illustrates a sample walking trial for AB01. The amputee subjects performed six sequences of three different walking modes, each lasting approximately 30 s.

In summary, we note a few important points. Firstly, in this paper, the type of walking activities used for recognition is not our main focus, but rather the assessment of the proposed methodology to eliminate irrelevant/redundant features for UIR is our main goal. Secondly, in human activity mode recognition applications, an entire stride is typically used for non-real-time classification [34]. However, in UIR, we use a small window of measurement signals, mostly within a few milliseconds, to identify user’s intent for real-time prosthesis control.

### 2.2. Data Windowing

To effectively classify human gait modes, we extract appropriate features from a frame (window) of measurement signals. $L_{f}$ is the length of a frame in milliseconds. The number of samples in the frame depends on the frame length $L_{f}$ and the sampling rate. A short frame fails to provide an informative data set and may lead to significant classification bias and variance. On the other hand, a long frame is a computational burden for real-time implementation. In this paper, $L_{f}$ is chosen to trade off feature informativeness and computational load.

**Figure 4:**
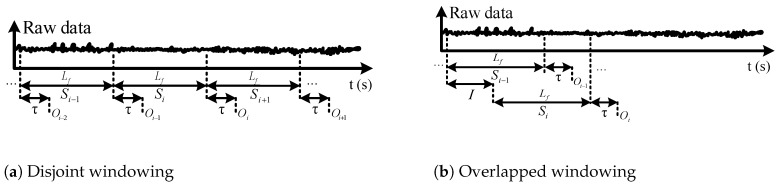
Data windowing. $S_{i}$ represents the *i*-th data segment, $L_{f}$ is the frame length, τ is the required processing time, *I* is the increment length for overlapped windowing, and $O_{i}$ is the detected gait mode corresponding to frame $S_{i}$.

We apply two different methods for data windowing: disjoint windowing and overlapped windowing [35]. Figure 4 illustrates the two windowing approaches. In disjoint windowing, the class outcome $O_{i}$ corresponding to frame $S_{i}$ is output every $L_{f}$ ms. τ is the time required for feature extraction, classification, commanding the appropriate low-level controller, and prosthesis response time. In overlapped windowing, we use a sliding frame with length $L_{f}$ and increment *I*, and the class outcome is output every *I* ms. Disjoint windowing is a special case of overlapped windowing when $I=L_{f}$. To achieve real-time operation, the parameters of the windowing approaches should satisfy

(1) $$\begin{array}{c} \begin{array}{c} \tau \leq L_{f}\;\;\;\;\;\;\;\;\;\;\;\;\mathrm{disjoint}\;\mathrm{windowing},\\ \tau \leq I\leq L_{f}\;\;\;\;\;\mathrm{overlapped}\;\mathrm{windowing}. \end{array} \end{array}$$

In this paper, we apply disjoint and overlapped windowing with various frame and increment lengths. We consider two important characteristics to determine $L_{f}$ [35]: (1) the minimum interval between two distinct muscle contractions is 200 ms [36], and (2) the delay between user intent and the resultant prosthesis motion should be no more than 300 ms [37,38]. The first property implies that a 200 ms frame of data should have the potential to provide informative features for gait mode classification. The second property, which is known as the real-time constraint, ensures that the amputee will experience the prosthesis as responsive to his or her intent. The real-time constraint requires $\tau \leq L_{f}\leq 300$ ms for disjoint windowing, and I≤300 ms for overlapped windowing. Therefore, we use overlapped windowing when the frame length is larger than 300 ms, noting that a larger frame will require a higher computational load.

### 2.3. Feature Extraction

Various features can be extracted from a frame of measurement data and used for classification. Features should be informative enough to discriminate between various gait modes. In addition, feature extraction needs to be computationally fast for real-time implementation. In general, both time-domain (TD) and frequency-domain (FD) features are frequently used for classification [35,39,40]. We compare TD and FD features in this paper, and select the optimal subset of features for UIR.

TD features are computationally fast, and include information about the data waveform and frequency. We extract the following TD features from each frame of data: slope sign change (SSC), zero crossing (ZC), waveform length (WL), variance (VAR), mean absolute value (MAV), modified MAV (MAV1 and MAV2), root mean square (RMS), Willison amplitude (WAMP), skewness (SK), kurtosis (KU), and correlation coefficient (COR) and angle (ANG) between two frames of data from different measurement signals. The mathematical definitions of these TD features are given in [39].

In addition, multiple FD features have been extracted. FD features are computationally slower than TD features, but include information about the frame’s frequency content. We use periodograms to measure the power spectrum density (PSD) of a frame, and calculate the following FD features: mean frequency (MNF), median frequency (MDF), maximum frequency (MAXF), and fourth-order auto-regressive coefficients (AR4). The mathematical definitions of these FD features are given in [39].

Previous research has shown the applicability of these TD and FD features for prosthetic limb pattern recognition [39,40,41]. Therefore, we are motivated to investigate the performance of these features for gait mode recognition.

After extracting TD and FD features from a frame of measurement data, the features are concatenated and labeled to create a single training pattern. For instance, extraction of VAR, MAV + RMS, and AR4 features from a frame of three measurement signals (e.g., vertical hip position, thigh angle, and thigh moment) would produce a training vector with 3, 6, and 12 elements, respectively. We perform the above procedure for all features and all frames of measurement data to create the training data set. The training set is then normalized to equalize the relative magnitude of each feature.

### 2.4. Feature Selection

The objective of feature selection is to find a subset of the features that were obtained with the feature extraction method. The feature selection method attempts to find a parsimonious feature subset that results in accurate classification. However, subset size and classification accuracy are conflicting objectives. A small feature subset will probably result in high classification error, whereas a large feature subset will probably result in lower classification error. Therefore, feature selection can be viewed as a multi-objective optimization (MOO) problem. In MOO problems, no single solution can simultaneously optimize all objectives. The solutions comprise a set of possible alternative solutions known as the optimal Pareto set [21].

We seek the most informative but parsimonious subset of features for gait mode classification. Note that exhaustive search is not practical in cases with a high-dimensional set of features. A set of *n* features has $2^{n}-1$ different subsets (excluding the null subset). Many heuristic search strategies, such as sequential forward selection, sequential backward elimination, and evolutionary search, have been suggested for this type of combinatorial problem [42]. Evolutionary algorithms (EAs) have been demonstrated as an efficient global search strategy for feature selection [43]. They generally outperform sequential forward selection and sequential backward elimination [22]. However, EA-based search strategies are computationally expensive due to the need for many cost function evaluations. To reduce computational complexity, we propose a new method called gradient-based multi-objective feature selection (GMOFS) for UIR. In addition, we propose the application of four EA-based search strategies, using multi-objective biogeography-based optimization (MOBBO), for feature selection. We then use two systematic approaches to compare the performance of the GMOFS search strategy with four variants of MOBBO.

### 2.5. Classification

Accurate classification of gait patterns is the ultimate goal of the user intent recognition (UIR) system. For this purpose, we assess various well-known linear and nonlinear classification techniques, including linear discriminant analysis (LDA), quadratic discriminant analysis (QDA), support vector machine (SVM), decision tree (DT), and multi-layer perceptron (MLP) classifiers.

LDA and QDA classifiers do not require time-consuming iterations for training. In fact, the parameters of these classifiers are directly obtained from the training data. Although these classifiers are fast in terms of training, they are not as flexible as nonlinear classifiers such as SVM, DT, and MLP. These classifiers solve an optimization problem that minimize the classification error. In most cases, it is difficult to find optimization solutions in closed form, so we use either gradient-based optimization algorithms such as steepest descent, or evolutionary algorithms (EAs).

We use one-against-one approach to implement multi-class SVM, and we also evaluate the performance of different kernels, such as linear and RBF. We tune the parameters of the SVM kernels to achieve the best classification performance. To increase the accuracy of the MLP network, we perform a grid search of the number of hidden nodes *p* from the set {3,4,5,6,8,10,15,20}, and we measure the mean classification error using five-fold cross validation (CV). Then, we choose *p* to obtain a trade-off between classification accuracy and classifier complexity. An MLP with small *p* may not result in the desired accuracy, but an MLP with large *p* may tend to memorize the noise in the training set and lead to overfitting and poor generalization. In addition, we use Wilcoxon signed-rank tests to statistically compare the classification methods.

### 2.6. Filter

To enhance the prediction performance of the UIR system, we incorporate a majority voting filter (MVF). MVF alleviates transient jumps between classifier output classes and leads to smooth transitions from one classified gait mode to another. We implement the MVF using 2q+1 classified modes [41]: the current, *q* previous, and *q* subsequent values. The MVF output is the most frequently classified mode among those 2q+1 values.

To obtain *q* subsequent classified gait modes, MVF enforces a time delay to allow the classifier to access the required data. The real-time constraint discussed in Section 2.2 requires a time delay less than 300 ms. Therefore, an appropriate value for *q* should be chosen to avoid violation of the real-time constraint. The constraint requires

(2) $$\begin{array}{c} \begin{array}{c} q\times L_{f}\leq 300\;\mathrm{ms}\;\;\;\;\;\;\;\;\mathrm{Disjoint}\;\mathrm{windowing},\\ q\times I\;\;\leq 300\;\mathrm{ms}\;\;\;\;\;\;\;\;\mathrm{Overlapped}\;\mathrm{windowing}. \end{array} \end{array}$$

An MVF with very small *q* may not significantly improve classification performance, whereas an MVF with very large *q* may cause misclassification because of time delay. In this paper, we will choose a trade-off value for *q*, and will investigate the effect of the MVF on classification performance.

## 3. Feature Selection Algorithm Development

In this section, we propose a search strategy based on biogeography-based optimization (BBO), in addition to a new gradient-based algorithm, to find an optimal subset of features for designing UIR with high accuracy and low complexity. We then use two systematic approaches to compare the performance of the different search strategies.

### 3.1. Biogeography-Based Multi-Objective Optimization

We propose the application of multi-objective biogeography-based optimization (MOBBO) for feature selection of the UIR system. The dimension of the optimization problem is equal to the number of available features (independent variables). Each feature is represented by a binary value where 1 indicates that the feature is used for classification, and 0 indicates otherwise. Therefore, each individual in the MOO algorithm is a binary sequence with length equal to the problem dimension. We evaluate the following two objective functions for all individuals in the population:

(3) $$\begin{array}{c} \begin{array}{c} f_{1}^{i}=\mathrm{number}\;\mathrm{of}\;\mathrm{selected}\;\mathrm{features}\mathrm{in}\;\mathrm{the}\;i-\mathrm{th}\;\mathrm{individual},\\ f_{2}^{i}=\mathrm{average}\;\mathrm{prediction}\;\mathrm{error}\;\mathrm{using}\;c-\mathrm{fold}\;\mathrm{cross}\;\mathrm{validation}, \end{array} \end{array}$$

where i=1,⋯,N, and *N* is the population size. We combine BBO with four MOO algorithms [21] to obtain vector evaluated BBO (VEBBO), non-dominated sorting BBO (NSBBO), niched Pareto BBO (NPBBO), and strength Pareto BBO (SPBBO). We apply these MOBBO variants to find the optimal Pareto set for the optimization problem. We investigate the performance of each method in a later section.

We use linear discriminant analysis (LDA) to compute $f_{2}^{i}$. LDA is widely used with evolutionary algorithms to evaluate the quality of a candidate subset for feature selection [44]. LDA does not require time-consuming iterations to build a model. This point is important because EAs require many objective function evaluations to find the solution. In Section 4.2, we will demonstrate that all feature selection approaches presented in this paper are able to find the most significant features, even though they use different selection criteria and machine learning methods. It is possible to use either classification accuracy or error as the quality measure for the second objective. We use average classification error of *c*-fold cross validation (CV). In *c*-fold CV, we randomly divide the training set into *c* distinct folds. Then, we repeat training *c* times; each time, the model is trained using c−1 folds and is tested with the remaining fold. The average of the *c* classification errors is used as the quality measure.

### 3.2. Gradient-Based Multi-Objective Feature Selection

Although MOBBO and other gradient-free MOO methods have the potential to find the globally optimal solution [22,23], they are computationally expensive due to the need for many iterations of the classifier training process (multiple individuals in the population, and multiple generations). To reduce computational complexity, we propose a novel algorithm called gradient-based multi-objective feature selection (GMOFS). In GMOFS, feature selection and data classification are performed simultaneously.

GMOFS incorporates a regularization penalty term to the optimization problem of its learning algorithm. The penalty term, which is handled by a Lagrange multiplier, directs the trained model toward a parsimonious as well as accurate model. We use an MLP network as the classifier, and include an elastic net to penalize the size of the selected feature subset. The first step of GMOFS is to train a constrained MLP network with the cost function

(4) $$J=\frac{1}{2}\sum_{l=1}^{m}\sum_{j=1}^{K}{\left(t_{j}^{\left(l\right)}-o_{j}^{\left(l\right)}\right)}^{2}+\lambda \sum_{i=1}^{n}\left(\alpha \beta_{i}^{2}+\left(1-\alpha \right)\left|\beta_{i}\right|\right),$$

where $\beta_{i}$ is the multiplier of the *i*-th input feature before input to the MLP network; $t_{j}^{\left(l\right)}$ and $o_{j}^{\left(l\right)}$ are the target and actual value, respectively, of the *j*-th output neuron associated with the *l*-th training pattern; *K* is the number of output neurons (classes); *m* is the number of training patterns; and *n* is the number of input features. We use an MLP network with one hidden layer and *p* hidden neurons (including the bias node). $v_{ih}$ denotes the weight that connects the *i*-th input neuron to the *h*-th hidden neuron, and $w_{hj}$ denotes the weight that connects the *h*-th hidden neuron to the *j*-th output neuron. The first term of the cost function penalizes classification error while the second term, which is the elastic net, penalizes the number of selected features. The elastic net is a convex combination of ridge regression (α=1) and least absolute shrinkage and selection operator (α=0). λ≥0 is a complexity parameter that controls the shrinkage of the input features. Large λ leads to a shrinkage of $\beta_{i}$ toward zero, which implies that the input feature corresponding to $\beta_{i}$ is not significant. However, as shrinkage increases, classification error tends to increase. Therefore, λ provides a trade-off between classification error and the number of selected features. In summary, the construction of the MLP network with the elastic net is formulated as the following optimization problem:

(5) $$\min_{\beta ,w,v}J\;\;\mathrm{subject}\;\mathrm{to}\;\;\left\{\begin{array}{c} 0\leq \beta_{i}\leq 1,\\ |w_{hj}\left|\leq a\;\mathrm{and}\;\right|v_{ih}|\leq b, \end{array}\right.$$

for all *i*, *j*, *h*, where $\beta_{i}=0$ or 1 implies that the associated feature is the least or most significant input variable, respectively. *a* and *b* are the bounds for neuron weights $w_{hj}$ and $v_{ih}$, respectively. However, due to the direct relationship between $\beta_{i}$ and neuron weights $v_{ih}$ and $w_{hj}$, we cannot conclude that an input feature with small $\beta_{i}$ and large neuron weights is insignificant. To avoid optimization solutions with large weights, neuron weights are constrained. Backpropagation is used to update $\beta_{i}$, $v_{ih}$, and $w_{hj}$. The derivative of *J* with respect to output weights $w_{hj}$, hidden weights $v_{ih}$, and input weights $\beta_{i}$ is obtained by the chain rule as

(6) $$\begin{array}{c} \begin{array}{cc} \frac{\partial J}{\partial w_{hj}} & =\sum_{l=1}^{m}\delta_{j}^{\left(l\right)}y_{h}^{\left(l\right)}\\ \frac{\partial J}{\partial v_{ih}} & =\sum_{l=1}^{m}\left[\sum_{k\in D_{2}\left(h\right)}^{}\left[\delta_{k}^{\left(l\right)}\;w_{hk}\right]y_{h}^{\left(l\right)}\left(1-y_{h}^{\left(l\right)}\right)\;\beta_{i}x_{i}^{\left(l\right)}\right]\\ \frac{\partial J}{\partial \beta_{i}} & =\sum_{l=1}^{m}\left\{\sum_{s\in D_{1}\left(i\right)}\left[\sum_{k\in D_{2}\left(h\right)}\left[\delta_{k}^{\left(l\right)}\;w_{sk}\right]y_{s}^{\left(l\right)}\left(1-y_{s}^{\left(l\right)}\right)v_{is}\right]x_{i}^{\left(l\right)}\right\}+\lambda \left[2\alpha \beta_{i}+\left(1-\alpha \right)\frac{\beta_{i}}{|\beta_{i}|}\right], \end{array} \end{array}$$

where $D_{1}\left(i\right)$ is the set of middle layer neurons whose inputs come from the *i*-th input neuron, $D_{2}\left(h\right)$ is the set of output neurons whose inputs come from the *h*-th middle layer neuron, and $\delta_{j}^{\left(l\right)}=-\left(o_{j}^{\left(l\right)}-t_{j}^{\left(l\right)}\right)\left(1-t_{j}^{\left(l\right)}\right)t_{j}^{\left(l\right)}$. Detailed derivation of the derivative of *J* with respect to $w_{hj}$ and $v_{ih}$ is available in [45]. The derivative of *J* with respect to input weights $\beta_{i}$ is straightforward to obtain using the chain rule. We use the derivatives in Equation (6) and constraints in Equation (5) along with the trust region reflective algorithm to train the constrained MLP network.

Once MLP training phase is completed, input weights $\beta_{i}$ are sorted in descending order. The input variable with the largest $\beta_{i}$ is the most significant feature. The second step of GMOFS is to select the most *r* significant features, which are associated with the *r* largest input weights $\beta_{i}$, and which satisfy

(7) $$\begin{array}{c} \begin{array}{c} \frac{\sum_{i=1}^{r}\beta_{i}}{\sum_{j=1}^{n}\beta_{j}}\geq 95\%,\\ \beta_{1}\geq \beta_{2}\geq \cdots \geq \beta_{n}. \end{array} \end{array}$$

The 95% threshold value in Equation (7) determines the trade-off between the number of selected features and the accuracy of the model. A low threshold value will decrease the number of selected features and will be more likely to remove informative features that can significantly contribute to the accuracy of the UIR model. On the other hand, a high threshold value will be more likely to include irrelevant features that cannot contribute to the accuracy of the model. To tune the threshold value, we gradually increase the threshold from zero, and, for each value, we compute the accuracies of the trained MLP once with the original $\beta_{i}$ values and once with $\beta_{i}=0$ for all unselected input features. We increase the threshold value until there is no significant difference between the performances of MLP for these two cases. That point was reached with the threshold value of 95%. We then repeat the first two steps of GMOFS for different λ in the range $\left[\lambda_{l},\lambda_{u}\right]$ with a predefined increment △λ. The selected subsets associated with each λ comprise a population. The population size depends on △λ. To assess the performance of the selected feature subset, we train a classifier with each selected subset and find classification error. In this population, the subset associated with λ→∞ has minimum size and maximum classification error, whereas the subset with λ=0 has maximum size and probably has the lowest classification error. Thus, the size of the selected subset and the classification error, defined in Equation (3), are two conflicting objectives. To find the GMOFS Pareto front, we first obtain the Pareto set as

(8) $$P_{s}=\left\{x^{*}:\left[∄x:f_{i}\left(x\right)\leq f_{i}\left(x^{*}\right)\;\mathrm{for}\;\mathrm{all}\;i\in \left[1,2\right],\;\mathrm{and}\;f_{j}\left(x\right)<f_{j}\left(x^{*}\right)\;\mathrm{for}\;\mathrm{some}\;j\in \left[1,2\right]\right]\right\}.$$

$x^{*}$ denotes the set of non-dominated solutions in the population and $f_{i}\left(x\right)$ is the *i*-th objective function. The Pareto front $P_{f}$ is obtained from all function vectors f(x) that correspond to the Pareto set:

(9) $$P_{f}=\left\{f\left(x^{*}\right):x^{*}\in P_{s}\right\}.$$

Note that all Pareto points are equally preferable apart from subjective prioritization. The outline of GMOFS is given in Algorithm 1.    

**Algorithm 1:** The outline of gradient-based multi-objective feature selection (GMOFS), where $x_{i}$ is the *i*-th feature in the training set *X*, and *Y* is the corresponding set of output classes.**Initialization:**$\lambda =\lambda_{l}\leq \lambda_{u}$, Population = ∅, k=1 **While**$\lambda \leq \lambda_{u}$   **Step 1:**   Use the training data {X,Y} to train the constrained MLP network in Equation (4)   by solving Equation (5)   **Step 2:**   Sort the input weights $\left\{\beta_{i}\right\}$ in descending order   Use Equation (7) to select subset $S_{k}\subset X$ where size($S_{k}$) ≤ size(*X*)   **Step 3:**   Population ← Population $+\;S_{k}$   k←k+1
**Next**
λ←λ+△λ
**Step 4:** **For** each subset $S_{k}$ in Population   Use cross-validation to train and test a classifier with dataset $\left\{S_{k},Y\right\}$   Calculate objective functions $f_{1}^{k}$ and $f_{2}^{k}$ using Equation (3) **Next** subset $S_{k}$
**Step 5:** Find the Pareto set using Equation (8)

### 3.3. Evaluation of Multi-Objective Optimization Pareto Fronts

We will compare the Pareto fronts obtained by each MOO algorithm using normalized hypervolume and relative coverage. These methods are popular for evaluating the quality of a Pareto front. The Pareto front normalized hypervolume is computed as follows:

(10) $$\mathrm{Normalized}\;\mathrm{Hypervolume}\;=\frac{\sum_{j=1}^{N_{p}}\prod_{i=1}^{M}f_{ji}}{N_{p}},$$

where *M* is the number of objectives, $f_{ji}$ is the value of the *i*-th objective function of the *j*-th Pareto point, and $N_{p}$ is the number of Pareto points.

Another way to compare Pareto sets is to compute the coverage of one Pareto set relative to a second Pareto set. This metric is determined by the number of solutions in the first Pareto set that are weakly dominated by at least one solution in the second Pareto set [21]. A smaller number for normalized hypervolume and relative coverage indicates better performance.

## 4. Results and Discussion

This section evaluates the performance of the user intent recognition (UIR) system and its subsystems as discussed in Section 2 and Section 3.

### 4.1. Effect of Frame Length on Classification Performance

The objective of this section is to choose the appropriate data windowing method and frame length. We investigate the influence of disjoint and overlapped windowing with different frame lengths on the classification accuracy of the UIR system. We use disjoint windowing with frame lengths $L_{f}=\left\{100,150,200,250\right\}$ ms, and overlapped windowing with frame lengths $L_{f}=\left\{200,250,300\right\}$ ms and increments I={50,150,200} ms. We extract time-domain (TD) and frequency-domain (FD) features from the data frames generated by the three measurement signals collected from able-bodied subjects. Note that the size of the data set depends on the parameters of the windowing methods. For instance, a 10-second walking sequence with disjoint windows of length 100 ms provides 100 frames and consequently 100 training patterns.

**Figure 5:**
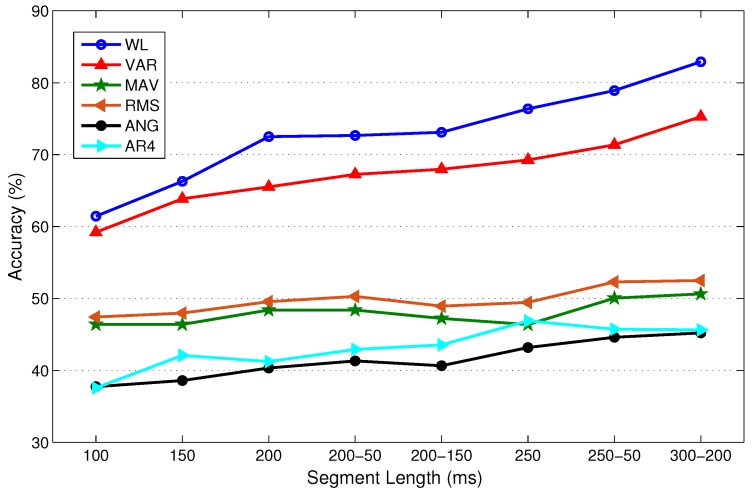
Mean LDA performance for the able-bodied subjects for different data windowing methods and frame lengths. On the horizontal axis, a single value indicates the frame length of disjoint windowing, and a pair of values indicates the frame length and increment length of overlapped windowing. For instance, 200–50 indicates $L_{f}=200$ ms and I=50 ms for overlapped windowing.

In this experiment, LDA, QDA, MLP, and SVM with RBF kernel are separately trained and tested using 10-fold cross validation (CV) for each subject with only one feature type for each of the measurements: WL, VAR, MAV, RMS, WAMP, ANG, and AR4. These features are used because they are considered the most representative TD and FD features. Figure 5 illustrates the mean classification accuracy of LDA with different frame lengths for able-bodied subjects using 10-fold CV. A single value on the horizontal axis of the figure indicates the frame length of disjoint windowing. A pair of values indicates the frame length and increment length of overlapped windowing; for instance, 200–50 denotes $L_{f}=200$ ms and I=50 ms. Figure 5 shows that the classification accuracy typically improves as the frame length increases. We observed similar trends with other classifiers. In fact, classifier type does not influence our choice of frame length. Therefore, we only provide classification results for LDA as a representative result.

Figure 5 shows that a larger frame is more likely to include more information, and consequently lower bias and variance in classification performance. For instance, the increase in accuracy with WL is approximately 18% when the frame length increases from 100 ms to 200 ms. The increases are 11.6% and 9.8% when using the VAR and AR4 features, respectively. However, the accuracy does not vary significantly with frame length for the remaining features in Figure 5. The figure illustrates that all representative TD features except ANG provide better classification performance than AR4, which is the representative FD feature.

In this experiment, very small frame length is not used because it would result in poor prediction accuracy. Conversely, large frame length is not used because it would result in a violation of the real-time constraint. To find the best frame length, we statistically compare performance using Wilcoxon signed-rank tests. For this purpose, LDA is trained multiple times, each time using one TD or FD feature type. We perform LDA training individually for each feature type rather than the full feature set to provide a sufficient number of samples for statistical comparison. The null hypothesis of the test is that the differences between mean classification accuracy (average accuracy of all LDAs trained individually with every single TD and FD feature type) corresponding to two different frame lengths are from a distribution with zero mean at the specified level of significance. If the null hypothesis cannot be rejected, then we conclude that the two compared frame lengths are not statistically significantly different, as indicated by an ≈ sign and a T (tie). If we can reject the null hypothesis, then the two frame lengths are statistically significantly different, and this is indicated by a + sign. The better frame length is the one with better mean classification accuracy and is shown by B (better) while the worse one is shown by W (worse). Table 2 provides the results of the statistical tests at a 10% significance level.

Table 2 shows that frames with length larger than 200 ms perform better than frames with length 150 or 100 ms. Table 2 shows that the two overlapped frame windows with $L_{f}=250$ ms, I=50 ms and $L_{f}=300$ ms, I=200 ms tie for similar performance, and perform better than the other frame lengths.

Taking into account the real-time constraint, the length of the MVF filter, and processing time, we choose overlapped windowing with $L_{f}=250$ ms, I=50 ms throughout the remainder of the paper as the best trade-off, except where specifically mentioned otherwise.

**Figure 6:**
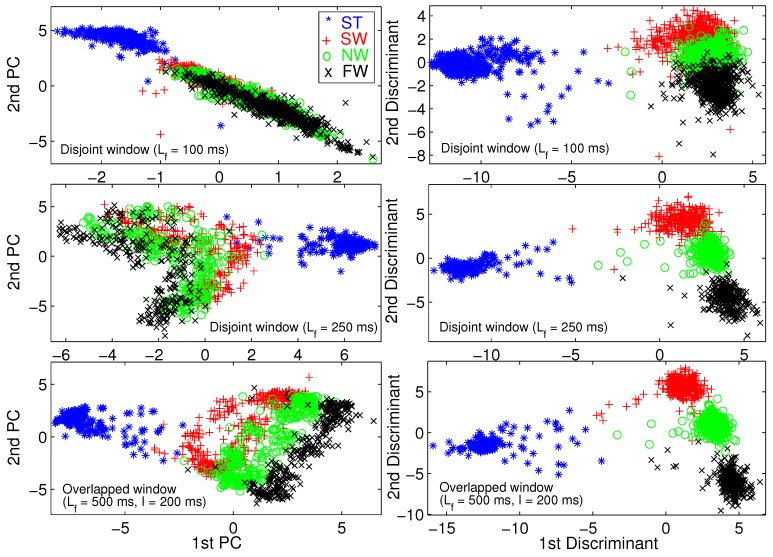
Two-dimensional scatter plot for visualization using principal component analysis (PCA) (**left column**) and Fisher linear discriminant analysis (FLDA) (**right column**) for able-bodied subject AB01.

We use principal component analysis (PCA) [46] and Fisher linear discriminant analysis (FLDA) [47] to visualize the training set. A training pattern is a vector of all TD and FD features extracted from a frame of raw data. We performed 2D dimension reduction for three different frame lengths. Figure 6 illustrates the 2D scatter plot for able-bodied subject AB01. To save space, we do not provide the same figures for other subjects because we obtained similar results for those subjects.

Figure 6 shows that FLDA provides better visualization than PCA in terms of gait mode separability. We verify that longer frame length leads to better gait mode separation, and eventually better classification performance. Most importantly, Figure 6 verifies the effectiveness of the TD and FD features.

### 4.2. Multi-Objective Feature Selection

We perform feature selection in two steps. In the first step, we exclude non-informative time-domain (TD) and frequency-domain (FD) features, and, in the second step, we use multi-objective optimization (MOO) to further refine the selected feature set. We implement five different MOO methods, including our newly proposed method, to find the optimal set of features. The complete set of features includes the following TD and FD features: $F_{1}$: slope sign change (SSC); $F_{2}$: zero crossing (ZC); $F_{3}$: waveform length (WL); $F_{4}$: variance (VAR); $F_{5}$: mean absolute value (MAV); $F_{6}$ and $F_{7}$: modified MAV (MAV1 and MAV2); $F_{8}$: root mean square (RMS); $F_{9}$: Willison amplitude (WAMP); $F_{10}$: skewness (SK); $F_{11}$: kurtosis (KU); $F_{12}$: median frequency (MDF); $F_{13}$: mean frequency (MNF); $F_{14}$: maximum frequency (MAXF); $F_{15}$ and $F_{16}$: correlation coefficient (COR) and angle (ANG) between two frames of data, respectively; and $F_{17}$: fourth-order auto-regressive coefficients (AR4). We perform preliminary feature selection using able-bodied subjects. We use MOO for final feature selection using only one able-bodied subject to reduce computational effort. In Section 4.4, we will investigate the performance of the selected features with other able-bodied subjects and with the amputee subjects. We note that the optimal feature subset may vary depending on subjects. However, in this paper, we are particularly interested in obtaining an optimal feature subset from able-bodied subjects, and assessing its performance on amputee subjects. The reason for this approach is that able-bodied subjects’ data are more accessible for UIR training in real-world applications. Our results in Section 4.4 will show no significant difference between UIR performances for amputee subjects when trained with the optimal feature subset and when trained with the full feature set. Future work could compare optimal subsets of features obtained from different individuals.

In the first step, we train LDA, QDA, SVM-Linear, SVM-RBF, and MLP for each of three able-bodied subjects, and separately for each individual feature type listed in the previous paragraph, using 10-fold cross-validation (CV) for each training procedure. The mean classification accuracy over the three subjects and the ten folds are used to assess the importance of each feature type.

**Figure 7:**
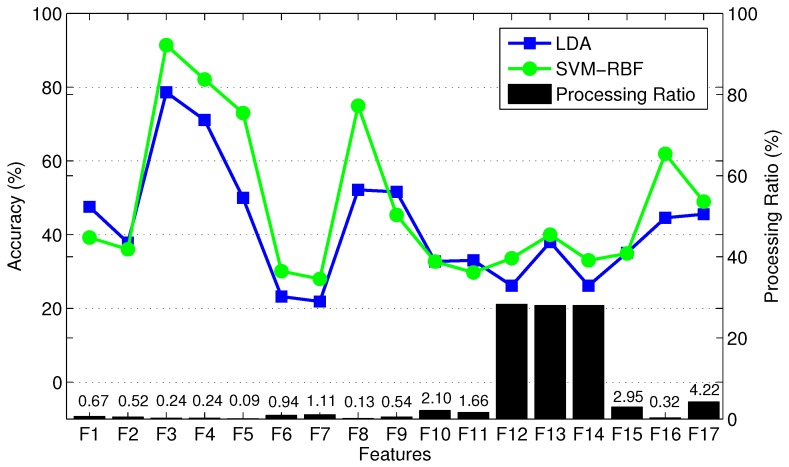
Mean classification accuracy of three able-bodied subjects, and processing ratio of 17 feature types trained by LDA using 10-fold cross validation.

Figure 7 shows the mean classification accuracy and processing ratio for each feature type. *Processing ratio* indicates the relative computational load to compute each feature type—for instance, the percentage of computational load required to compute $F_{17}$ over the computational load required to compute all feature types is 4.22%. To reduce clutter in the figure, we show LDA results as a representative of QDA and SVM-Linear since they had similar performance. Similarly, we show SVM-RBF as a representative of MLP. Figure 7 shows consistent performance of different classifiers in terms of prioritizing various feature types. Figure 7 indicates that TD features require less computational effort than FD features. For instance, $F_{12}$, $F_{13}$, and $F_{14}$ require high computational effort compared to other features. We exclude $F_{12}$, $F_{13}$, and $F_{14}$ from the candidate feature set due to their relatively high computational expense and poor classification accuracy. In addition, $F_{6}$ and $F_{7}$, two variants of MAV, are excluded due to their poor classification accuracy and because they provide information that is similar to MAV. Therefore, we exclude a total of five weak feature types, and pre-select the remaining 12 feature types. This results in a training vector with 11×3+4×3=45 elements. Note that the AR4 feature type includes four components and thus contributes a total of 12 elements from the three measurement signals. Finally, vertical hip position does not cross zero (see Figure 3), thus the number of zero crossing (ZC) feature of this signal is excluded. To verify the lack of information in the eliminated features, we found that the combination of the excluded features with the pre-selected features did not significantly enhance classification performance. In summary, we have a training data set with 44 features.

Now, we are ready to proceed to the feature selection step. In this step, we use vector evaluated BBO (VEBBO), non-dominated sorting BBO (NSBBO), niched Pareto BBO (NPBBO), strength Pareto BBO (SPBBO), and gradient-based multi-objective feature selection (GMOFS) to select an optimal subset from the 44 pre-selected features. To reduce the computational expense, we use only the AB01 training data set in this step. We then verify that the selected subset results in a satisfactory UIR system when trained for other subjects. Table 3 shows the tuning parameters used in this paper. To tune the parameters, we performed a sensitivity analysis of multi-objective optimization (MOO) performance to each parameter, one at a time, to find a local optimum of MOO performance with respect to each parameter. For instance, GMOFS is implemented with different elastic net parameter values α={0,0.5,1}, and we found that the Pareto front with α=0 dominates Pareto fronts that are found with other values of α. For training the neural network in GMOFS, we used the MATLAB function fmincon from the Optimization Toolbox (R2014a, MathWorks, Natick, MA, USA) to implement a trust region reflective algorithm. We mostly used default values for the fmincon parameters, but we found that the performance of GMOFS is not very sensitive to these parameters.

We run each multi-objective method for 10 independent trials, and the best Pareto front of each method is selected for MOO comparison. Results show that the GMOFS Pareto front statistically significantly dominates all four multi-objective biogeography-based optimization (MOBBO) Pareto fronts. We note that GMOFS and the MOBBO variants use different classifiers for feature selection, namely, MLP and LDA. To obtain a fair comparison of the new components of GMOFS with the MOBBO variants, we decouple the search strategy from classification performance. Note that LDA, which is used in MOBBO, is one of the most popular classification algorithms and has been widely used with evolutionary algorithms for feature selection due to its good performance and simplicity [44].

**Figure 8:**
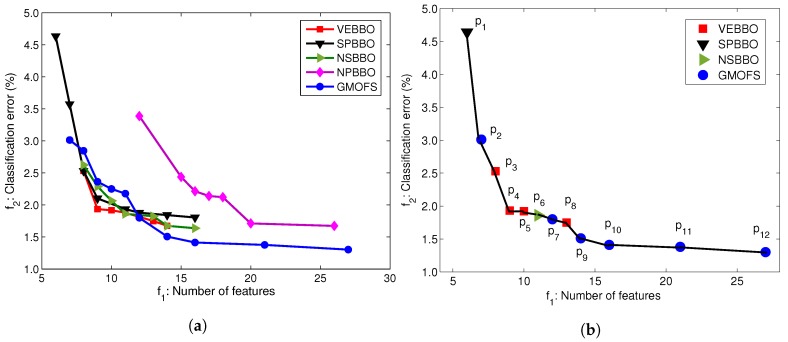
(**a**) Pareto fronts obtained from MOO methods with an SVM classifier with linear kernels using AB01 training data; (**b**) combined Pareto front obtained from non-dominated Pareto points in (**a**).

To conduct the fair comparison, we apply SVM with linear kernels to all of the optimal feature subsets found by the MOO methods. Figure 8a illustrates the Pareto fronts obtained by the five MOOs with SVM with linear kernels. Figure 8a shows that the Pareto fronts of VEBBO, SPBBO, NSBBO, and GMOFS are close, and clearly dominate the NPBBO Pareto points. Figure 8b indicates the combined Pareto front obtained from all of the non-dominated points in Figure 8a. GMOFS provides the maximum contribution to the combined Pareto front, while NPBBO does not contribute any Pareto points. All of the points in Figure 8b are labeled for easy referencing.

To systematically compare the Pareto fronts in Figure 8a, we use relative coverage and normalized hypervolume as discussed in Section 3.3. Table 4 and Table 5 provide the comparison results using these two approaches. In Table 4, an entry in column *i* and row *j* (i≠j) indicates the percentage of Pareto points of the method of column *i* that is dominated by at least one Pareto point of the method of row *j*. We see that, on average, only 7.2% of the Pareto points of VEBBO are weakly dominated by at least one Pareto point from the other four MOO methods. Therefore, VEBBO ranks first in terms of relative coverage. GMOFS ranks second and performs better than SPBBO, NSBBO, and NPBBO. In addition, Table 5 shows that VEBBO and GMOFS rank first and second in terms of normalized hypervolume, respectively. GMOFS ranks first in terms of the number of Pareto points. These results verify the competitive performance of GMOFS compared to the other four MOO methods.

Most importantly, in terms of the advantage of GMOFS, it requires the execution of only 43 classifier training procedures (due to the number of λ increments), while each of the other four EA-based MOO methods require 100,000 training procedures (due to the combination of population size and generation limit).

The benefit of presenting the data of Figure 8b is that it allows us to find the best subset of features for an accurate and parsimonious classifier. Among the 12 Pareto points, we choose $p_{9}$ as a potential candidate solution. We could pick any other solution from the Pareto front depending on the priority of the problem objectives, but $p_{9}$ provides a good trade-off between classification error and number of features. Therefore, in Section 4.4, we will investigate classification performance with candidate solution $p_{9}$ for all human subject data AB01, AB02, AB03, AM01, AM02, and AM03. However, first, we will find the best classifier in the following section.

**Figure 9:**
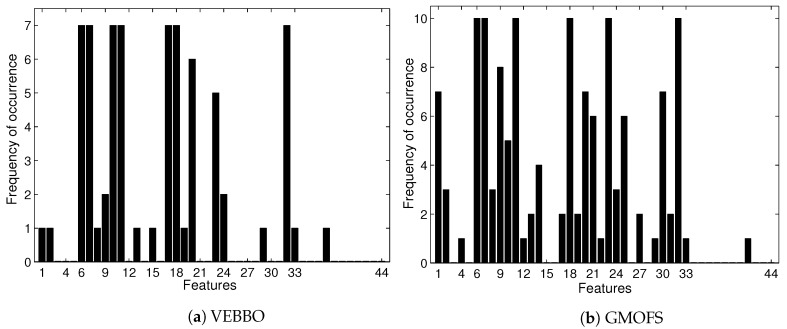
Selection frequency of 44 features by VEBBO and GMOFS. The plots show how many times each feature appears in the Pareto points of the given method. For instance, feature 6 is present in all 10 GMOFS Pareto points.

Figure 9 shows that the feature selection frequencies of GMOFS and VEBBO, taken across all Pareto points, are different. However, they both select significant features at a high frequency. There are five common features that appear in all of the GMOFS, VEBBO, NSBBO, NPBBO, and SPBBO Pareto points: WL from vertical hip position and thigh angle (features 6 and 7), VAR from thigh moment (feature 11), WAMP from vertical hip position (feature 18), and ANG from thigh moment (feature 32). Therefore, all five feature selection methods value the most informative features regardless of their selection criterion and machine learning method. For example, Pareto point $p_{8}$ (obtained by VEBBO combined with the LDA classifier) and candidate solution $p_{9}$ (obtained by the GMOFS combined with MLP classifier) have nine features in common out of a total of 13 and 14 features, respectively.

### 4.3. Comparison Results of Classification Algorithms

In this section, we use $p_{1}$ through $p_{12}$ to statistically compare the performance of different classifiers for subject AB01. The objective is to find the best classifier for locomotion mode detection among linear discriminant analysis (LDA), quadratic discriminant analysis (QDA), support vector machine (SVM) with linear kernels (SVM-linear), SVM with RBF kernels (SVM-RBF), multi-layer perceptron (MLP), and decision tree (DT). The tuned parameter value of RBF kernel is σ=1. Table 6 shows mean classification accuracy and standard deviation of each classifier trained with the features from each Pareto point using 10-fold cross validation (CV).

Table 7 presents pairwise statistical comparisons using Wilcoxon signed-rank tests at a 5% significance level. If a pairwise *p*-value is less than 0.05, the mean performances of the two classifiers are statistically significantly different, and the classifier with larger mean prediction accuracy performs better than the other one. A pairwise *p*-value greater than 0.05 indicates no significant difference between the performance of the two classifiers. Table 7 shows that the classification performance of MLP and SVM-RBF are statistically equal, and are significantly better than the other methods. SVM-linear is statistically better than LDA, QDA, and DT. QDA performs better than LDA and similarly to DT. In summary, MLP and SVM-RBF are the best, SVM-linear is the second best, QDA and DT are the third best, and LDA is the worst for locomotion mode detection.

### 4.4. Performance Assessment of Selected Subset

In this section, we evaluate UIR for all able-bodied and transfemoral amputee subjects with feature subset $p_{9}$. All classifiers are trained with three representative methods (SVM-RBF, SVM-linear, and QDA). The RBF kernel tuning parameter is σ=1 and σ=4 for able-bodied and amputee subjects, respectively. In this section, we use multiple-fold CV to train and test UIR, where each walking sequence that consists of different gait modes is considered a fold (see Section 2.1). In training phase, we use all walking sequences except one to train UIR. We then test the UIR accuracy on the excluded walking sequence (fold). Accuracy is defined as the total number of correctly classified test patterns divided by the total number of test patterns. We repeat training and testing by shifting the excluded folds. We calculate the accuracy averaged over all folds to find the mean performance of UIR.

We use average classification error of *c*-fold cross validation (CV). In *c*-fold CV, we randomly divide the training set into *c* distinct folds. Then, we repeat training *c* times; each time, the model is trained using c−1 folds and is tested with the remaining fold. The average of the *c* classification errors is used as the quality measure.

We saw in Section 2.2 that overlapped windowing with frame length $L_{f}=250$ ms and increment I=50 ms is the best data window option. For real-time operation, a conservative choice for parameter q=5 satisfies the constraint q×I≤300 ms. Therefore, we use a majority voting filter (MVF) with length 2×q+1=11. Results verify a fast processing time on a standard desktop computer of less than 50 ms, on average, including feature extraction and classification with each of the three classifiers.

**Figure 10:**
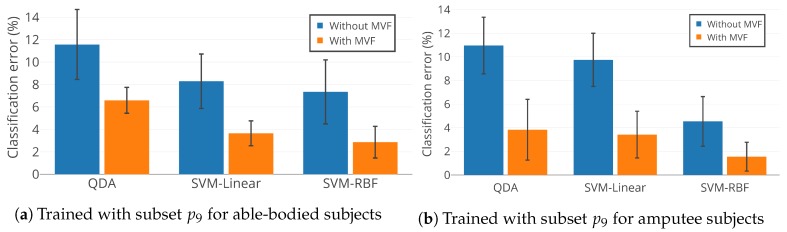
Classification performance of QDA, SVM-Linear, and SVM-RBF with feature subset $p_{9}$ for able-bodied subjects (AB01, AB02, and AB03) and amputee subjects (AM01, AM02, and AM03).

Figure 10 illustrates the mean classification error of QDA, SVM-linear, and SVM-RBF trained with feature subset $p_{9}$. Training was conducted individually for able-bodied subjects AB01, AB02, and AB03 and amputee subjects AM01, AM02, and AM03, with and without MVF. Figure 10 indicates that: (1) SVM-RBF outperforms SVM-linear and QDA, which confirms the statistical results in Section 4.3; (2) MVF statistically significantly decreases classification error for locomotion mode detection (p<0.05); and (3) $p_{9}$ is an effective feature subset and results in accurate as well as compact UIR. Feature subset $p_{9}$ uses only 14 features out of a total of 60 available features, which reduces the size of the feature set by 77%.

SVM-RBF was also trained for the able-bodied subjects with the full set of 60 features. When combined with MVF, it results in a mean classification accuracy of 98.54%±1.92%. In comparison, we achieve 97.14%±1.51% mean classification accuracy with feature subset $p_{9}$, which includes only 14 features. Statistical tests at 5% significance level indicate no significant difference between UIR performance when trained with the full feature set and subset $p_{9}$.

SVM-RBF was also trained for the amputee subjects with the full set of 60 features. When combined with MVF, it results in a mean classification accuracy of 99.37%±0.96%. In comparison, we achieve 98.45%±1.22% mean classification accuracy with feature subset $p_{9}$. As with the able-bodied subjects, statistical tests indicate no significant difference between UIR performance when trained with the full feature set and subset $p_{9}$. This indicates the satisfactory performance of our framework, which is able to eliminate unneeded features with no significant degradation in overall accuracy.

In this paper, we decoupled the optimization problem of window length and feature selection by dividing it into two smaller sequential optimization problems [35]. We may obtain a suboptimal feature subset with this approach, but this point is not critical since we were able to find accurate and simple UIR that has no meaningful performance difference than UIR designed with the full feature set.

## 5. Conclusions

We presented a framework for designing a UIR system. We used experimental data collected from three able-bodied subjects and three above-knee amputee subjects to classify four and three different gait modes, respectively. Overlapped windowing with frame length 250 ms and increment 50 ms provided a good trade-off between classification performance and real-time computation. Several efficient TD and FD features were extracted from data frames to form the feature set. We performed feature selection in two steps. First, we excluded non-informative features with poor classification performance and high computational effort. Second, we used MOO to find an optimal feature subset from the remaining features to obtain a UIR system that was both parsimonious and accurate. For this purpose, GMOFS, a novel embedded multi-objective feature selection algorithm, was proposed and compared with four evolutionary MOOs on the basis of normalized hypervolume and relative coverage. Classification results confirmed the competitive performance of GMOFS. Several classifiers were trained with the optimal feature subsets that were selected by MOO, and SVM-RBF and MLP were found to be the best classifiers for UIR. The outputs of the classifiers were input to an MVF to improve classification accuracy and chattering between the identified classes.

For future work, more above-knee amputee subjects will be involved in data collection and classification. In addition, we will include other daily-life activities such as incline walking, stair ascent and descent, standing and sitting, etc. It is also of great interest to consider other informative features for classification, such as wavelet transform coefficients. Finally, it would be of interest to compare GMOFS with other state-of-the-art MOO methods, and to apply GMOFS to other MOO problems.

## Figures and Tables

**Table 1:** Physical characteristics of the six human test subjects. AB and AM represent able-bodied and amputee subject, respectively.

	Gender	Age	Weight	Height	Walking Speed (m/s)		
		(years)	(kg)	(cm)	SW	PS	FW
AB01	Male	37	79.5	188	0.98	1.30	1.63
AB02	Male	20	73.9	172	0.86	1.15	1.44
AB03	Male	28	80.9	179	0.75	1.00	1.25
AM01	Male	32	79.1	174	0.60	1.00	-
AM02	Male	64	99.2	177	0.56	0.94	-
AM03	Male	35	81.7	176	0.60	0.90	-

**Table 2:** Comparison of mean classification performance for different frame lengths (row values versus column values) using Wilcoxon signed-rank tests at a 10% significance level. Mean classification performance is considered as the average of all linear discriminant analysis (LDA) classifiers trained individually with every single time-domain (TD) and frequency-domain (FD) feature type. ≈ indicates that the two compared frame lengths tie (T) with similar performance and are not statistically significantly different. + indicates that the two frame lengths are statistically significantly different, and B or W indicates that the row frame length performs better or worse than the column frame length, respectively. * indicates that the lower triangular half of the table is equal to its upper triangular half.

Frame Length (ms)		150	200	200–50	200–150	250	250–50	300–200
100	vs.	W (+)	W (+)	W (+)	W (+)	W (+)	W (+)	W (+)
150	vs.	−	W (+)	W (+)	W (+)	W (+)	W (+)	W (+)
200	vs.	*	−	W (+)	T (≈)	W (+)	W (+)	W (+)
200–50	vs.	*	*	−	T (≈)	T (≈)	W (+)	W (+)
200–150	vs.	*	*	*	−	W (+)	W (+)	W (+)
250	vs.	*	*	*	*	−	W (+)	W (+)
250–50	vs.	*	*	*	*	*	−	T (≈)

**Table 3:** Tuning parameters for multi-objective feature selection.

	Symbol	Value
**MOBBO**		
Mutation rate	μ	0.04
Number of elites	*E*	2
Population size	*N*	100
Number of generations	Gen	1000
Problem dimension	*d*	44
Migration model	$m_{\mathrm{flag}}$	sinusoidal
**GMOFS**		
Number of hidden nodes	*p*	5
Elastic net parameter	α	0
Bound for shrinkage parameter	[$\lambda_{l},\lambda_{u}$]	[0, 150]
Bound for neuron weights	a,b	5
Increment of shrinkage parameter	△λ	1 if 0≤λ≤30; and 10 if 30<λ≤150
**Trust region reflective**		
Maximum allowable iterations	MaxIter	100
Termination tolerance on the independent variable	TolX	0.001
Termination tolerance on the cost function	TolFun	0.001
Typical values for the independent variable	TypicalX	0.1
Finite difference method	FinDiffType	central

**Table 4:** Comparison of Pareto fronts using relative coverage (RC). Only 7.2% and 30% of the VEBBO and GMOFS points, respectively, are dominated by other Pareto points; so VEBBO and GMOFS rank first and second, respectively, in terms of RC.

	VEBBO	SPBBO	NSBBO	NPBBO	GMOFS
VEBBO	−	62.5	75.0	85.7	40.0
SPBBO	0.0	−	25.0	71.4	40.0
NSBBO	14.3	50.0	−	100.0	40.0
NPBBO	0.0	0.0	0.0	−	0.0
GMOFS	14.3	50.0	50.0	100.0	−
Mean RC (%)	7.2	40.4	37.5	89.3	30.0

**Table 5:** Comparison of Pareto fronts using normalized hypervolume. $N_{p}$ is the number of Pareto points obtained by each MOO method. VEBBO and GMOFS rank first and second, respectively, in terms of normalized hypervolume, and GMOFS ranks first in terms of the number of points.

	$N_{p}$	Normalized Hypervolume
VEBBO	7	0.5026
SPBBO	8	0.5814
NSBBO	8	0.5676
NPBBO	7	0.8013
GMOFS	10	0.5332

**Table 6:** Mean classification accuracy (ACC) and standard deviation (STD) for AB01 of classifiers trained with 13 different feature subsets. NF is the number of features in each set.

Pareto Point	NF	LDA		QDA		SVM-Linear		SVM-RBF		MLP		DT	
		ACC	STD	ACC	STD	ACC	STD	ACC	STD	ACC	STD	ACC	STD
$p_{1}$	6	93.56	0.740	94.33	0.852	95.37	1.218	98.33	0.421	97.34	0.698	96.15	1.16
$p_{2}$	7	95.31	0.829	96.06	0.711	96.99	0.775	98.88	0.216	98.29	0.539	96.35	1.00
$p_{3}$	8	96.69	0.835	96.82	0.410	97.47	0.694	98.86	0.378	98.20	0.411	96.67	1.18
$p_{4}$	9	96.86	0.684	96.95	0.484	98.07	0.576	99.31	0.234	98.34	0.459	96.73	1.25
$p_{5}$	10	97.04	0.657	96.99	0.427	98.08	0.430	98.90	0.406	98.47	0.645	96.56	1.22
$p_{6}$	11	96.84	0.536	97.15	0.654	98.14	0.692	98.94	0.293	98.62	0.578	96.32	1.28
$p_{7}$	12	96.93	0.656	97.36	0.372	98.20	0.497	99.05	0.356	98.87	0.406	97.21	1.00
$p_{8}$	13	96.61	0.384	97.62	0.554	98.25	0.534	99.14	0.305	95.76	9.180	96.86	1.36
$p_{9}$	14	96.76	0.485	97.79	0.311	98.49	0.500	98.88	0.290	98.90	0.355	97.15	0.78
$p_{10}$	16	96.95	0.525	97.84	0.578	98.59	0.467	98.38	0.449	98.66	0.371	96.99	0.72
$p_{11}$	21	97.13	0.501	97.93	0.716	98.62	0.564	98.40	0.392	99.00	0.432	97.30	0.54
$p_{12}$	27	97.41	0.568	97.77	0.861	98.70	0.422	97.58	0.663	99.07	0.373	96.91	0.64

**Table 7:** Comparison of classification performance using Wilcoxon signed-rank tests (W.T.) at a 5% significance level. B or W indicates that the row method performs better or worse than the column method, respectively, while T shows that they tie with similar performance. * indicates that the lower triangular half of the table is equal to its upper triangular half. These results are obtained using all the data from Table 6.

		DT		SVM-RBF		SVM-linear		QDA		LDA	
		*p*-Value	W.T.	*p*-Value	W.T.	*p*-Value	W.T.	*p*-Value	W.T.	*p*-Value	W.T.
MLP	vs.	2.44 × 10${}^{-4}$	B	7.32 × 10${}^{-1}$	T	8.50 × 10${}^{-3}$	B	5.02 × 10${}^{-3}$	B	2.44 × 10${}^{-4}$	B
DT	vs.	−		1.23 × 10${}^{-4}$	W	8.20 × 10${}^{-3}$	W	1.33 × 10${}^{-1}$	T	1.70 × 10${}^{-1}$	T
SVM-RBF	vs.	*		−		6.70 × 10${}^{-3}$	B	2.44 × 10${}^{-4}$	B	1.22 × 10${}^{-4}$	B
SVM-linear	vs.	*		*		−		1.15 × 10${}^{-4}$	B	1.25 × 10${}^{-4}$	B
QDA	vs.	*		*		*		−		2.44 × 10${}^{-4}$	B

## Abbreviations

The following abbreviations are used in this manuscript.

List of acronyms in order of appearance			
**Acronym**	**Definition**	**Acronym**	**Definition**
UIR	User intent recognition	ZC	Zero crossing
MOO	Multi-objective optimization	WL	Waveform length
GMOFS	Gradient-based multi-objective feature selection	VAR	Variance
MOBBO	Multi-objective biogeography-based optimization	MAV	Mean absolute value
SVM	Support vector machine	RMS	Root mean square
RBF	Radial basis function	WAMP	Willison amplitude
MVF	Majority voting filter	SK	Skewness
sEMG	Surface electromyography	KU	Kurtosis
LDA	Linear discriminant analysis	COR	Correlation
QDA	Quadratic discriminant analysis	ANG	Angle
GMM	Gaussian mixture model	PSD	Periodogram spectrum density
ANN	Artificial neural network	MNF	Mean frequency
BBO	Biogeography-based optimization	MDF	Median frequency
VEBBO	Vector evaluated BBO	MAXF	Maximum frequency
NSBBO	Non-dominated sorting BBO	AR	Auto-regressive model
NPBBO	Niched Pareto BBO	CV	Cross validation
SPBBO	Strength Pareto BBO	AB01	Able-bodied subject 01
EA	Evolutionary algorithm	AM01	Amputee subject 01
MLP	Multilayer perceptron	PS	Preferred speed
TD	Time domain	ST	Standing
FD	Frequency domain	NW	Normal walking
FLDA	Fisher’s linear discriminant analysis	SW	Slow walking
PCA	Principal component analysis	FW	Fast walking
DT	Decision tree		
SSC	Slope sign change		
